# A Feasibility Randomized Controlled Trial of a Parenting Intervention Offered to Women With Severe Mental Health Problems and Delivered in a Mother and Baby Unit Setting: The IMAgINE Study Outcomes

**DOI:** 10.3389/fpsyt.2022.815018

**Published:** 2022-05-16

**Authors:** Anja Wittkowski, Richard Emsley, Penny E. Bee, Elizabeth Camacho, Rachel Calam, Kathryn M. Abel, Paula Duxbury, Paula Gomez, Kim Cartwright, Holly E. Reid

**Affiliations:** ^1^Division of Psychology and Mental Health, School of Health Sciences, Faculty of Biology, Medicine and Health, The University of Manchester, Manchester, United Kingdom; ^2^Department of Clinical Psychology, Laureate House, Wythenshawe Hospital, Greater Manchester Mental Health NHS Foundation Trust, Manchester, United Kingdom; ^3^Manchester Academic Health Science Centre, The University of Manchester, Manchester, United Kingdom; ^4^Department of Biostatistics and Health Informatics, Institute of Psychiatry, Psychology and Neuroscience, King's College London, London, United Kingdom; ^5^Division of Nursing, Midwifery and Social Work, School of Health Sciences, Faculty of Biology, Medicine and Health, The University of Manchester, Manchester, United Kingdom; ^6^Division of Population Health, Health Services Research and Primary Care, Faculty of Biology, Medicine and Health, School of Health Sciences, The University of Manchester, Manchester, United Kingdom; ^7^Department of Research and Innovation, Greater Manchester Mental Health NHS Foundation Trust, Manchester, United Kingdom

**Keywords:** mothers, intervention, perinatal, severe mental illness (SMI), parenting, inpatient admission, feasibility and acceptability

## Abstract

**Background:**

Approximately 1–2% of mothers may experience severe mental illness (SMI) requiring admission to an inpatient Mother and Baby Unit (MBU). MBUs aim to provide mental health assessment and treatment and strengthen the mother-infant relationship, essential for infant development. Whilst MBUs offer various interventions, they do not routinely offer structured parenting interventions. The Baby Triple P Positive Parenting Program (BTP) was developed to enhance parenting competence, psychological coping and the quality of partner and other social support. Guided by lived experience consultation, we aimed to determine the feasibility and acceptability of delivering BTP plus Treatment as Usual (TAU) in this setting.

**Method:**

A multi-site, parallel-group, single-blind pilot randomized controlled trial (registration: ISRCTN12765736) comparing BTP+TAU to TAU in participants, recruited from two MBUs in England. The Baby Triple P intervention consisted of eight parenting sessions, with the final four being delivered over the telephone following MBU discharge. Feasibility outcomes were participant intervention engagement and study retention. Clinical outcomes including maternal parenting competence, bonding and mental health outcomes were assessed at baseline, post-baseline/intervention (10 weeks) and six-month follow-up. Data were analyzed using descriptive statistics and linear regression models. An economic feasibility analysis was also conducted.

**Results:**

Thirty-seven of the 67 eligible participants consented; 34 were randomized (16 to BTP+TAU and 18 to TAU), of whom 20 were retained at post-intervention data collection and 21 at six-month follow-up. Twelve participants (75%) completed the intervention, which was rated as highly acceptable. Clinical outcomes signaled potential improvements in maternal parenting competence, bonding, mood and mental health symptomatology in participants who received the intervention. Healthcare resource use and EQ-5D-5L questionnaires were well-completed by participants. Delivering BTP in this setting is estimated to cost £443-822 per participant.

**Conclusions:**

This is the first trial of a parenting intervention in a MBU setting. BTP is feasible and acceptable to mothers with SMI, with a promising signal for treatment efficacy. Although minor modifications may be required for the collection of observer-rated measures post-MBU discharge, the findings indicate that a larger, definitive trial could be conducted, especially if the setting is extended to include perinatal mental health community settings.

## Introduction

Approximately 10–20% of women develop mental health difficulties during pregnancy or the first year of having a baby, with an estimated 1% experiencing severe mental illness requiring specialist psychiatric services (1–3). Severe mental illness (SMI) in the perinatal period refers to severe and incapacitating depression, psychosis, schizophrenia, bipolar disorder, schizoaffective disorder and postpartum psychosis (2, 3). Within the literature, it has long been established that maternal mental illness has a significant, detrimental impact on the woman, her family, and her developing child [e.g., (4–10)]. The quality of the mother-infant interaction in women experiencing SMI or severe mental health difficulties can be poorer compared to mothers experiencing affective disorders [e.g., (11, 12)]. For example, Wan et al. (11) observed mothers with schizophrenia to be less responsive and sensitive toward their infants and the infant in turn were more avoidant.

Mother and Baby Units (MBUs) offer an inpatient setting for the treatment of mothers experiencing severe mental illness in the perinatal period whereby mothers are admitted jointly with their babies [e.g., (1, 2, 13)]. However, despite improvements in mental health in mothers admitted to these units, research has found that the treatment of the mother's symptoms does not necessarily always translate into more positive and attuned interactions with her baby (12). Early interventions may be a good solution to promote nurturing environments and parent and baby interaction because the potential benefits of these interventions in the general population have been largely accepted, especially in relation to child outcomes (14–19).

The Triple P system of interventions is a major contribution to the parenting intervention research with solid theoretical, scientific and clinical foundations (20–25). Triple P interventions are aimed at contributing to the healthy development of children by enhancing parental knowledge and resourcefulness regarding positive parenting practices. Given the substantial long-term negative effects of early adverse experiences, and the capacity of positive relationships to buffer or modulate these effects, an expansion of Triple P interventions has resulted in an intervention to address parental practices and needs in families expecting a baby (23). Baby Triple P is a positive parenting intervention aimed at preparing parents for their transition into parenthood by providing them with knowledge and skills to promote secure attachment with a new baby, to improve the quality of partner support alongside wider social support and to increase coping resources to reduce parental distress (26).

First-time parents rated this universal intervention to be acceptable (27). There is also evidence from studies with non-psychiatric parents that Baby Triple P has reduced infant distress in terms of inconsolable crying in six-month-old infants who also appeared to be more content in contrast to infants in the control group (28). At 2 years of corrected age, pre-term children were also found to have significantly better cognitive function, motor and symbolic communication skills compared to their control group (29). Furthermore, the acceptability and feasibility of Baby Triple P was explored in a sample of mothers (76.9% were primiparous) with postnatal depression in order to see if it could be beneficial within a mental health context (30). Of the 27 women randomized to treatment as usual or the intervention in this pilot trial, all 12 women who received Baby Triple P rated the intervention as highly acceptable and all of them were retained until the final follow up. Although this study was underpowered for analysis of effect, the results were in the predicated direction post-intervention in terms of reported levels of depression, happiness, self-regulation and subjective bonding. Acceptability and applicability in a more severe mental illness context were further examined in two related studies, using Q-methodology to explore the views and attitudes of mothers with SMI admitted to a MBU as well as MBU staff (31, 32). Mothers believed that a parenting intervention like Baby Triple P would be beneficial to them. They also deemed the MBU environment to be suitable for its delivery (31). This view was shared by MBU staff who also endorsed that this type of intervention would be feasible within the MBU setting and acceptable to mothers, regardless of their personal situation (32).

The preventive focus of the Baby Triple P programme on strengthening the mother and baby relationship or bond as well as on reducing maternal stress and increasing social support could be beneficial, specifically for women presenting with perinatal mental health problems. Despite the availability of psychiatric interventions for mothers experiencing severe mental health difficulties, no structured parenting interventions are routinely offered within these specialist perinatal settings.

The aim of this study was to evaluate the acceptability of the Baby Triple P parenting intervention in mothers with severe mental health problems in a MBU setting and the feasibility of recruiting, engaging and retaining women in this study with a view to evaluating it in a full-scale randomized controlled trial (33). In particular, this study aimed to (1) establish the suitability and acceptability of the study procedures for mothers experiencing severe mental health problems admitted to a Mother and Baby Unit, (2) determine whether there were any signals that the intervention might improve maternal and infant outcomes and (3) identify key drivers of cost associated with the intervention.

## Methods

### Design and Study Setting

This study used a multisite, parallel-group single-blind (outcome assessors) randomized controlled trial (RCT) design to compare Baby Triple P plus treatment as usual (BTP+TAU) with TAU only in a MBU setting, with one MBU located in the Northwest (Site 1) and one in the Midlands (Site 2), in the UK. These MBUs had a capacity of 10 and 9 beds, respectively, and were comparable in serving a largely urban and ethnically and socio-economically diverse group of female service users and their families.

A mixed-methodology approach was used to establish the feasibility and acceptability of the intervention and study procedures [see (33), for the study protocol]. Acceptability was explored in depth through participant and MBU staff interviews and these qualitative findings will be published elsewhere (34).

### Ethical Approvals and Research Governance

This trial was supported by the NIHR Research for Patient Benefit Programme (NIHR RfPB, grant number PB-PG-1014-3505) and sponsored by The University of Manchester. Study approvals were granted by the NHS National Research Ethics Service (NRES) *via* the Northwest–Greater Manchester South Research Ethics Committee (REC) (16/NW/0510), the Health Research Authority (HRA) (IRAS project number 188486, protocol number 16233) and the Research and Innovation departments of both NHS trusts overseeing the two participating MBUs (Greater Manchester Mental Health NHS Foundation Trust and Birmingham and Solihull Mental Health NHS Foundation Trust). Furthermore, an independent Trial Steering and Data Monitoring Committee and a Patient and Public Involvement (PPI) group supported this study through regular meetings throughout the feasibility trial's duration.

### Participant Inclusion and Exclusion Criteria

Women jointly admitted with their babies to one of the two participating MBUs were screened against the following eligibility criteria: Participants had to: (1) be aged ≥18 years, (2) have at least one infant aged <12 months or be in the third trimester of pregnancy and expected to reside on the MBU following delivery and (3) be proficient in English to provide written informed consent and/or participate in the study assessments, interviews and, if allocated, in the intervention.

Participants were not eligible for this study if they had any of the following characteristics: (1) they experienced significant psychiatric symptoms that compromised their ability to concentrate on assessments or intervention sessions, (2) they showed severe personality disorder traits including self-harming behaviors or (3) their infants were removed from their care on a non-temporary basis. Participants were also excluded from study participation if their discharge from the MBU was scheduled within seven days of them expressing interest in the study because they would be unable to complete the initial four sessions within a week if randomized to the intervention.

### Recruitment

Recruitment was conducted until April 2018 (commencing November 2016 at Site 1 and from March 2017 at Site 2). Recruitment methods involved MBU staff identifying participants who met the eligibility criteria and were willing to be approached by the research team to receive information about the study. A “Consent to Approach” form was used to document potential participants' consent to be contacted by a member of the research team. Each participant provided written, informed consent and their continued consent was sought regularly by the project manager prior to each assessment. The full recruitment procedure is detailed in Wittkowski et al. (33).

### Randomization

Participants were asked to complete the first set of outcome measures at baseline before they were randomly allocated to either BTP+TAU or TAU only. The randomization list was held by the Manchester Academic Health Science Centre Clinical Trials Unit (MAHSC-CTU), subsequently known as the Manchester CTU. The allocation ratio was 1:1 with randomized permuted blocks of size 4 and 6.

### The Baby Triple P Intervention

The BTP programme consisted of eight sessions, which were delivered by trained facilitators: a clinical psychologist at Site 1 and an occupational therapist at Site 2. A full description of the sessions is presented in Table 1. Participants allocated to receive this intervention were given the BTP workbook (26) to keep. They were advised to share it with their partners and, if desired, other family members, but not to share it with other mothers on the MBU to avoid contamination of outcomes. All participants adhered to this request.

**Table 1:** Session content summary of the baby triple P positive parenting programme.

**Session number/Theme**	**Content covered in session**	**Strategies**
Session 1—Positive parenting	• Aims of positive parenting. • Factors that impact child development • Strategies for promoting healthy development. • Strategies to promote secure attachment and healthy interactions with baby. • Goal setting for first 12 months as a parent.	Communication strategies to show affection to baby.
Session 2—Responding to your baby	• Responding to baby • Teaching of new behaviors and skills	Praising baby, show attention, providing interesting/novel activities and setting routines.
Session 3—Survival skills	• Identification of unpleasant emotions and how they affect parenting. • Identification of unhelpful ways to think about parenting (parenting traps) • Expectations of transition to parenthood. • Common experiences when having a new baby.	Coping skills, settling techniques, relaxation and stress management techniques, establishing boundaries, coping plans development, though identification, social support.
Session 4—Partner support	• Common experiences in couples in transition to parenthood. • Identification of unhelpful ways of thinking about relationship. • Communication skills for maintaining relationship wellbeing.	Communication, constructive feedback, support for each other, problem solving approach, sharing task and activities.
Sessions 5 to 8—Implementing parenting routines	• Prompting self-evaluation, • Goal-setting and planning for areas of future change. • Identifying obstacles and risks and strategies to address them.	All as indicated above.

*Adapted from Tsivos et al. (30)*.

### Intervention Fidelity and Process Evaluation

Both facilitators were trained by an accredited BTP trainer to deliver the intervention, which was supported by a facilitator manual. In addition, the facilitators recorded a log of the amount of their time spent on delivering the intervention. To ensure fidelity of the intervention delivery, both facilitators also completed BTP specific checklists following each session and discussed intervention delivery and its challenges in regular peer assisted supervision and support sessions [for further details of this supervision model, see (35, 36)]. Furthermore, five sessions were digitally recorded and assessed by an independent and experienced Triple P therapist and supervisor, who confirmed that BTP sessions were delivered with high content fidelity and high process quality.

### Treatment as Usual (TAU)

TAU consisted of case management using a care programme approach provided by allocated MBU psychiatric staff including consultant psychiatrists, nurses and nursery nurses and pharmacological interventions as well as non-parenting psychological interventions (e.g., CBT for depression). TAU varied according to patient needs, MBU capacity and staff availability, but excluded any parenting interventions. The variability of psychosocial and psychological interventions offered in MBUs in the UK has been documented elsewhere [see (37, 38)]. As both MBUs admitted women from anywhere in England and Wales based on bed availability, post-discharge care varied and depended on local service provision. Hence, TAU following MBU discharge included multidisciplinary team management offered by perinatal community mental health teams (CMHTs), where available, or by crisis or home treatment teams, non-perinatal CMHTs or Improving Access to Psychological Therapies (IAPT) teams.

### Outcome Measures

The primary outcome was the feasibility of recruiting and retaining participants to the study and the intervention. The feasibility of BTP delivery was assessed *via* engagement with the intervention (i.e., percentage of sessions attended) and acceptability were derived from participants' satisfaction with the intervention, which was assessed *via* the *Client Satisfaction Questionnaire* (CSQ) (39).

Secondary outcomes, collected to identify signals of effectiveness and key drivers of cost-effectiveness, are summarized in Table 2. The suitability and acceptability of outcome measures were informed by data completeness analysis (i.e., number of items responded by active participants). Outcome data were collected by research assistants blind to the allocation arm at three time points during the study: Time 1 (baseline), Time 2 (10 weeks post-baseline) and Time 3 (6 months post-baseline).

**Table 2:** Overview of outcome measures used.

**Outcome measure**	**What is being measured**	**Score interpretation**	**Completed by**	**Time 1**	**Time 2**	**Time 3**
*Family Background Questionnaire* [FBQ, (40)] and *Maternal Social Support* (41)	Sociodemographic characteristics including social support	FBQ—n/a, mostly descriptive MSS: higher scores indicate better perceived social support. Cut off scores suggest <18=low, 19–24 = medium and >24 = adequate levels of support	Participants	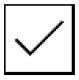		
*Maternal Efficacy Questionnaire* [MEQ, (42)]	Maternal self-efficacy	Higher scores indicating higher maternal efficacy	Participants	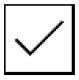	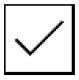	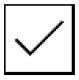
*Brief Depression, Anxiety and Stress Scale* [DASS-21, (43–45)]	Subjective mood and stress	Higher scores indicate worse mood or higher stress Cut off scores are: 1. 0–4 = normal, 5–6 = mild, 7–10 = moderate, 11–13 = severe and ≥14 extremely severe levels of depression 2. 0–3 = normal, 4–5 = mild, 6–7 = moderate, 8–9 = severe and ≥10 = extremely severe levels of anxiety 3. 0–7 = normal, 8–9 = mild, 10–12 = moderate, 13–16 = severe and ≥17 extremely severe levels of stress	Participants	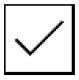	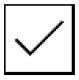	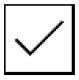
*Brief Symptom Inventory* [BSI, (46)]	Psychiatric symptom presence and severity	Scores exceeding 63 indicate clinical significance and increased psychopathology.	Participants	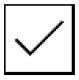	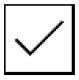	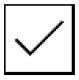
*Postpartum Bonding Questionnaire* [PBQ, (47, 48)]	Subjective mother-baby relationship and bond	Lower scores indicate better perceived bonding, and higher scores indicate poorer bonding and higher maternal psychopathology. Cut of scores are: 1. <11 = high bond and ≥12 low bond (PBQ Impaired Bonding) 2. <16 = normal mother-infant relationships and scores from 17–35 indicate high mother-infant relationship disorders (PBQ Rejection and Pathological Anger) 3. 1–9 = low infant-focused anxiety and ≥10 = high infant-focused anxiety (PBQ Infant-focused Anger) 4. 1–2 = low maternal pathological anger and ≥3 = high maternal pathological anger (PBQ Incipient Abuse)	Participants	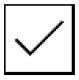	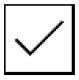	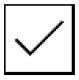
*Five-Level EQ-5D* [EQ-5D-5L, (49–51)]	Health status, used to calculate quality adjusted life years (QALYs)	All five dimensions have five response levels. Lower scores indicate better health and higher scores indicate worse health	Participants	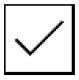	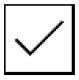	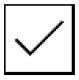
*Health and Social Care Resource Use Questionnaire*	Capturing resource use during the study period	N/A	Participants		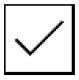	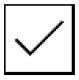
*Clinical Global Impression Scale* [CGI, (52)]	Improvement from admission to discharge	This scale includes 3 factors (severity of mental illness, improvement since admission, efficacy of treatment with medication compared to severity of side effects). High and low scores indicate worse and better mental health respectively. Score interpretations state: 1. Minimum score = 1 (normal), maximum score = 7 (among the most extremely ill patients) (Severity of mental illness) 2. Minimum score = 1 (very much improved), maximum score = 7 (very much worse) (Improvement since admission) 3. Minimum score = 0 (marked improvement, no side-effects), maximum score = 4 (unchanged/ worse, side effects outweigh therapeutic effects). (Efficacy of treatment with medication compared to severity of side effects)	MBU staff	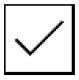	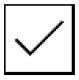	Could no longer be rated by MBU staff
*Brief Psychiatric Rating Scale [BPRS, (53, 54)]*	Psychiatric symptom severity	7-point Likert scale for 18 factors (minimum score = 18, maximum score = 126). Lower scores indicated better mental health and higher scores indicated worse mental health. Cut-off scores state: 18–31= mildly ill, 32–41= moderately ill, 42–53= markedly ill, >53 = severely ill.	MBU staff	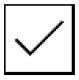	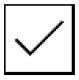	Could no longer be rated by MBU staff
*Louis MACRO (Mother and Child Risk Observation) Measure* (55)	Infant wellbeing and mother-baby-relationship	Higher scores indicate better status and lower risk.	MBU staff	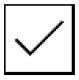	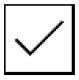	Could no longer be rated by MBU staff

### Procedure

Full details of the procedure are reported elsewhere (33). After consenting to the study, participants completed the baseline assessment measures and MBU staff were asked to complete relevant observer-rated measures. Participants were randomly allocated to continue with TAU alone or to receive the intervention in addition to TAU during their MBU admission. Participants allocated to the intervention were usually offered weekly sessions. At each site, the project manager, who was not blind to the allocation, offered session reminders to participants and checked ongoing consent prior to each follow up assessment. These assessments were typically conducted in participants' homes because most participants were discharged from the MBU by this stage. All relevant health and safety procedures were followed.

After study completion, all participants were offered £30 as a reimbursement for their time and contributions alongside a certificate of completion and a list of useful contacts or organizations for additional support. After the last follow-up assessment was completed, participants in the TAU only condition were also offered the BTP workbook.

### Data Analysis

The statistical and economic analyses were described in the protocol (33) and follow the intention-to-treat principle. Analyses were conducted in STATA (56) and SPSS (57).

We report participant flow using the CONSORT Statement for Pilot and Feasibility Studies (58), and descriptive summaries were generated for the outcome measures. A linear regression model was used to estimate the effect of treatment allocation on the self-reported outcomes at post-intervention (i.e., 10 weeks post-baseline, Time 2) and at 6-month follow-up (Time 3) separately, adjusting for outcome measures at baseline. Adjusted mean differences, bias corrected bootstrap standard errors and 95% confidence intervals (CIs) are reported as well as Cohen's *d* standardized effect sizes (calculated from the adjusted mean differences and the pooled standard deviation at baseline) and their corresponding 95% CIs.

Resource use data were collected from an NHS and social care perspective. The currency used was GBP (£) and price year 2018. Descriptive summaries were generated for the EQ-5D-5L and healthcare resource use data. Utility values were derived using the crosswalk methodology (50) as currently recommended by NICE (51) and quality adjusted life years (QALYs) were calculated using an area under the curve approach. The cost of delivering the Baby Triple P intervention was estimated based on the number of hours that facilitators reported spending on delivering the intervention, and the respective unit cost (59) of the NHS grade that each facilitator was employed on. The cost of training the facilitators, based on the employment grade of the person who delivered the training and duration of training, was also included.

## Results

### Participant Characteristics

The two groups of participants were comparable across most demographic and clinical characteristics (see Table 3) and reflect the wider urban MBU population as well. On average participants were 29 years old and their partners slightly older at 33 years old. Their infants, of which 55% were girls, were mostly the result of planned (59%) but complicated (62%) pregnancies. The infants had an average age of 14.62 (SD = 10.31) weeks. The mothers were mostly British (68%), primiparous (59%), married or cohabiting (61% and 29%, respectively) and rated their partner and wider social support to be moderate (20.81, SD = 3.41).

**Table 3:** Demographic, psychiatric, psychosocial and delivery-related characteristics of the participants, their infants and partners.

	**Total (*n =* 34)**	**BTP + TAU** **(*n =* 16)**	**TAU Only (*n =* 18)**
**Maternal characteristics**			
Mean age (years) (SD)	29.3 (4.1)	29.3 (0.98)	29.3 (4.4)
Perceived severity of current psychological difficulties (M and SD, from a 1 to 10 scale)	6.5 (1.66)	6.7 (1.6)	6.3 (1.6)
Currently taking medication (yes%)	97% (*n =* 33)	94% (*n =* 15)	100% (*n =* 18)
Mean length of stay in MBU in weeks (SD)	9.2 (4.5)	9.5 (5.2)	9.1 (3.9)
Mental health history - previous psychological difficulties (yes%)	85% (*n =* 29)	87% (*n =* 14)	83% (*n =* 15)
Affective disorders (% of sample with previous mental health difficulties)	13% (*n =* 4)	–	26% (*n =* 4)
Affective disorders + anxiety (% of sample with previous mental health difficulties)	72% (*n =* 21)	86% (*n =* 12)	60% (*n =* 9)
Other (% of sample with previous mental health difficulties)	6% (*n =* 2)	–	13% (*n =* 2)
Did not specify (% of sample with previous mental health difficulties)	10% (*n =* 2)	14% (*n =* 2)	–
Difficulties occurring during previous pregnancies (yes, % of sample with previous mental health difficulties)	17% (*n =* 5)	14% (*n =* 2)	20% (*n =* 3)
**Psychiatrist diagnosis**			
Postpartum psychosis	12% (*n =* 4)	6% (*n =* 1)	16% (*n =* 3)
Bipolar disorder	15% (*n =* 5)	19% (*n =* 3)	11% (*n =* 2)
Depression with psychotic features	12% (*n =* 4)	6% (*n =* 1)	16% (*n =* 3)
Depression	21% (*n =* 7)	31% (*n =* 5)	11% (*n =* 2)
Anxiety (including GAD and PTSD)	6% (*n =* 2)	12% (*n =* 2)	0% (*n =* 0)
Anxiety (including GAD, PTSD and OCD) and affective disorders	21% (*n =* 6)	12% (*n =* 2)	16% (*n =* 4)
Personality disorder and affective disorders	15% (*n =* 5)	6% (*n =* 1)	16% (*n =* 4)
Schizophrenia	2% (*n =* 1)	6% (*n =* 1)	0% (*n =* 0)
**Infant characteristics**			
Mean age (weeks)	**14.6 (10.3)**	**13.6 (2.5)**	**15.5 (2.5)**
Gender (Female %)	55% (*n =* 19)	63% (*n =* 10)	50% (*n =* 9)
**Relationship status (%)**			
Married	61.8% (*n =* 21)	50% (n = 8)	72% (*n =* 13)
Living together	29.4% (*n =* 10)	50% (*n =* 8)	11% (*n =* 2)
Single	8.8% (*n =* 3)	0% (*n =* 0)	17% (*n =* 3)
**Parity (%)**			
First time parent	59% (*n =* 20)	63% (n = 10)	50% (*n =* 10)
Two children	29% (*n =* 10)	37% (n = 6)	20% (*n =* 4)
Three children	6% (*n =* 2)	–	11% (*n =* 2)
Over three children	6% (*n =* 2)	–	11% (*n =* 2)
**Ethnicity (%)**			
British	68% (*n =* 24)	75% (*n =* 12)	61% (*n =* 11)
Other white background	18% (*n =* 6)	13% (*n =* 2)	20% (*n =* 4)
Asian British	12% (*n =* 4)	6% (*n =* 1)	16% (*n =* 3)
Other mixed background	3% (*n =* 1)	6% (*n =* 1)	–
**Education level (%)**			
No qualifications	6% (*n =* 2)	6% (*n =* 1)	6% (*n =* 1)
GCSEs, CSEs or O-levels	18% (*n =* 6)	19% (*n =* 3)	17% (*n =* 3)
A levels/BTEC	18% (*n =* 6)	19% (*n =* 3)	17% (*n =* 3)
Trade/apprenticeship	15% (*n =* 5)	25% (*n =* 4)	6% (*n =* 1)
University degree	24% (*n =* 8)	25% (*n =* 4)	22% (*n =* 4)
Postgraduate degree	9% (*n =* 3)	–	17% (*n =* 3)
Other	12% (*n =* 4)	6% (*n =* 1)	17% (*n =* 3)
**Family income (%)**			
Upper-middle – High	38% (*n =* 13)	44% (*n =* 7)	33% (*n =* 6)
Middle	38% (*n =* 13)	38% (*n =* 6)	39% (*n =* 7)
Low-middle – Low	24% (*n =* 8)	19% (*n =* 3)	28% (*n =* 5)
Reported financial issues in the last 12 months	15% (*n =* 5)	13% (*n =* 2)	28% (*n =* 5)
**Maternal employment (%)**			
Full-time	9% (*n =* 3)	–	17% (*n =* 3)
Part-time	6% (*n =* 2)	6% (*n =* 1)	6% (*n =* 1)
Home-duties	15% (*n =* 5)	–	28% (*n =* 5)
Maternal leave	50% (*n =* 17)	63% (*n =* 10)	39% (*n =* 7)
Unemployed	21% (*n =* 7)	31% (*n =* 5)	11% (*n =* 2)
**Partner characteristics**			
Mean partner/husband age (years)	33 (6.8)	33 (7)	32 (6.6)
Previous diagnosis of depression (yes)	3% (*n =* 1)	6% (*n =* 1)	–
**Partner's education (%)**			
No qualifications	3% (*n =* 1)	6% (*n =* 1)	–
GCSEs, CSEs or O-levels	24% (*n =* 8)	13% (*n =* 2)	33% (*n =* 6)
A levels/BTEC	12% (*n =* 4)	13% (*n =* 2)	11% (*n =* 2)
Trade/apprenticeship	15% (*n =* 5)	25% (*n =* 4)	6% (*n =* 1)
University degree	24% (*n =* 8)	25% (*n =* 4)	22% (*n =* 4)
Other	24% (*n =* 8)	31% (*n =* 5)	17% (*n =* 3)
**Partner employment (%)**			
Full-time	68% (*n =* 23)	88% (*n =* 14)	50% (*n =* 9)
Part-time	6% (*n =* 2)	–	11% (*n =* 2)
Home-duties	6% (*n =* 2)	–	11% (*n =* 2)
Unemployed	15% (*n =* 5)	13% (*n =* 2)	17% (*n =* 3)
**Pregnancy characteristics (%)**			
Planned pregnancy (yes %)	59% (*n =* 20)	63% (*n =* 10)	53% (*n =* 10)
Complications during pregnancy (yes %)	62% (*n =* 21)	56% (*n =* 9)	67% (*n =* 12)
Vaginal delivery	53% (*n =* 18)	50% (*n =* 8)	53% (*n =* 10)
Induced labor	21% (*n =* 7)	19% (*n =* 3)	20% (*n =* 4)
**Assisted delivery**			
Forceps	15% (*n =* 5)	25% (*n =* 4)	5% (*n =* 1)
Ventouse	3% (*n =* 1)	–	5% (*n =* 1)
Episiotomy	12% (*n =* 4)	13% (*n =* 2)	10% (*n =* 2)
Fetal Monitoring	12% (*n =* 4)	6% (*n =* 1)	17% (*n =* 3)
Emergency Cesarean	12% (*n =* 4)	13% (*n =* 2)	10% (*n =* 2)
Planned Cesarean	18% (*n =* 6)	19% (*n =* 3)	17% (*n =* 3)
Other	3% (*n =* 1)	–	5% (*n =* 1)
**Maternal social support (mean/SD)**	20.8 (3.4)	20.4 (4.9)	20.6 (4.2)
Feels supported by friends	3.6 (1.3)	3.5 (1.3)	3.7 (1.3)
Feels supported by family	4.1 (1.2)	4.3 (1.2)	3.9 (1.3)
Feels supported by husband/partner	4.5 (0.9)	4.7 (0.5)	4.2 (1.1)
Experiences high level of conflict with husband/partner	3.4 (1.0)	3.5 (1.0)	3.2 (1.2)
Feels being controlled by husband/partner	4.0 (1.5)	4.5 (1.1)	3.6 (1.8)
Feels loved by husband/partner	4.2 (1.2)	4.4 (1.0)	4.0 (1.4)

*Values in bold indicate significant differences between groups at p < 0.05*.

Although infants in the TAU group were on average 2 weeks older than those in the BTP+TAU group at baseline, there were no other differences between the two groups which suggests that randomization was performed successfully.

In terms of their mental health, most participants reported previous mental health difficulties (85%) and were currently taking medication (97%), predominantly for affective diagnoses with anxiety (72%). The most common diagnoses were depression (21%) and anxiety with an affective disorder (21%) but only 6% received a diagnosis of an anxiety disorder only. The other most common diagnoses were bipolar disorder (15%) and personality disorder with affective disorder (15%). Postpartum psychosis was diagnosed in 12% of the participants. A higher percentage of participants from the TAU only group received a diagnosis of postpartum psychosis (16%) and of personality disorders with affective disorders (16%) compared to the BTP+TAU group (6 and 6% respectively). Further diagnostic and psychosocial information can be found in Table 3 and Supplementary Table 1.

### Changes to the Study Protocol

As the original grant submission for this study predated the publication of this CONSORT statement, we analyzed the data in accordance with our original funding submission and published protocol [see (33)]. However, there were some changes to the original protocol (see Supplementary Table 2 for full details). Based on preliminary data on the capacity (19 mothers and babies per unit per month) and turnover [average admission duration of approximately seven weeks; (37)] of the two participating MBUs, we anticipated a potential pool of 209 women to be admitted and aimed to recruit approximately 60 to this feasibility trial. During the study recruitment period, however, the admission rate was considerably lower, with women being admitted for longer with an average of about nine weeks. As the amount of available data was low, analyses were conducted using all available values rather than performing multiple imputation. NHS site was not included as a covariate along with group and baseline measures, due to the low number of participants recruited at Site 2. Due to the low number of participants assessed at post-baseline with observer-rated measures, regression analysis was also not conducted using observer-rated measures, except for the CGI (52). Originally, research staff were going to complete observer-rated measures, but during the study the research team and trial steering committee agreed that, despite training in questionnaire administration, research assistants lacked the expertise of clinical psychiatric and nursing staff to adequately rate symptoms of mental health. Finally, as one of the sites had stopped administering the Health of the Nation Scale (60) as a routine outcome measure, it was also not used in any analyses.

Although two blind breaches per site occurred, in all four cases it was possible for another trained assessor to undertake these assessments instead. Hence, research assistants who undertook data collection remained blind to the participants' allocation arm.

### Feasibility of Recruitment and Retention

**Figure 1:**
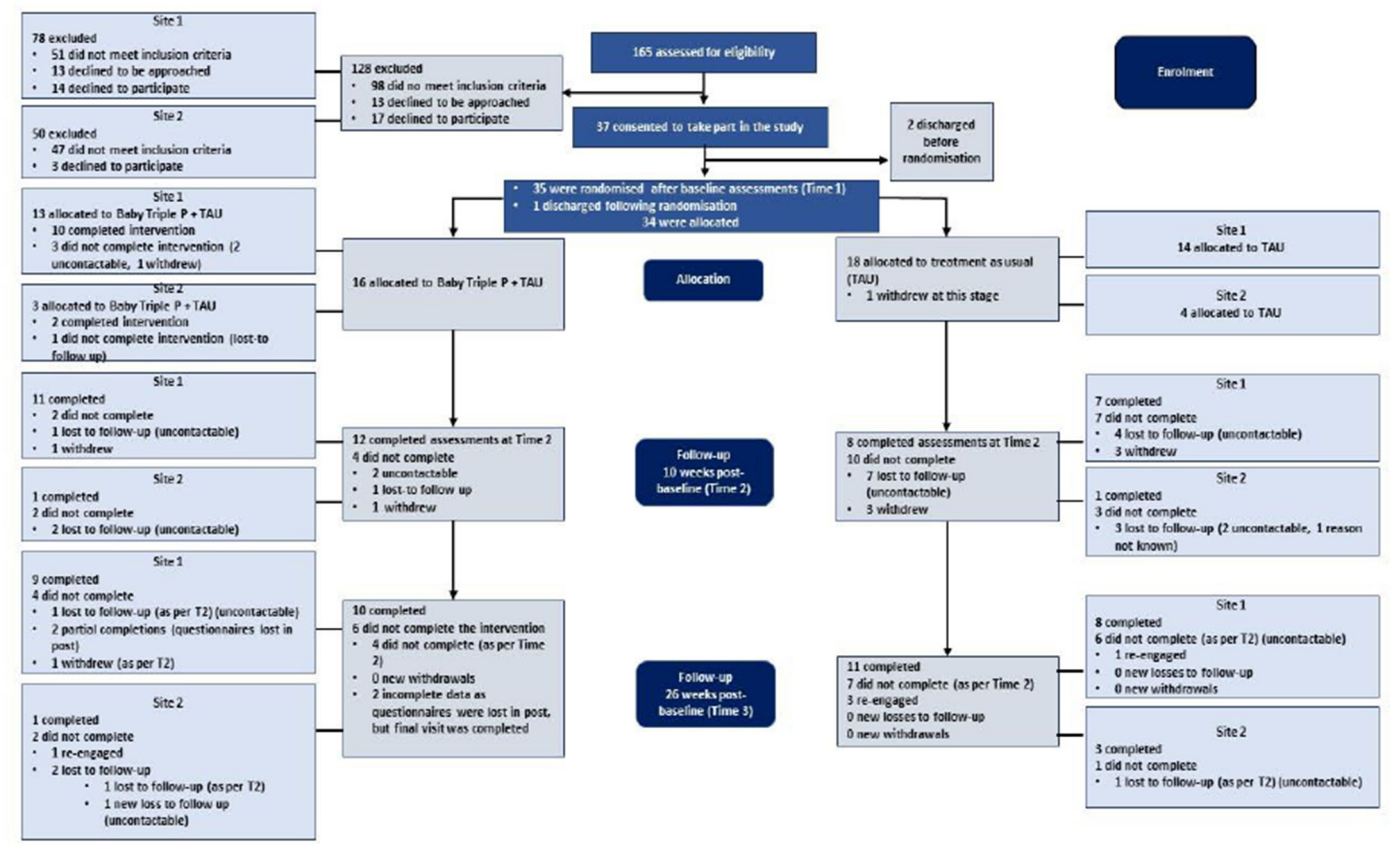
Consort diagram.

The flow of participants through the study is summarized in Figure 1. All 165 women (100%) admitted to one of the two MBUs during the recruitment period were screened, but 98 (59%) were not eligible. Of the 67 eligible women, 37 (55%) consented to take part in the study. However, two participants were discharged before randomization took place and one participant was discharged following randomization, making all three ineligible. The remaining 34 participants (roughly 52% of the 65 eligible participants) were randomized to receive either TAU only (*n* = 18) or BTP+TAU (*n* = 16). As can be seen in Figure 1, a greater number of participants were recruited at Site 1 (*n* = 27) than at Site 2 (*n* = 7).

In terms of study retention, 21 of the 34 eligible participants (approximately 62%) completed the final follow-up assessment, but retention rates varied considerably between the groups from baseline to the two assessment timepoints (e.g., 75 and 63% for BTP+TAU and 44–61% for TAU). Only four participants from the BTP+TAU group did not complete assessments at Time 2, in contrast to 10 from the TAU only group. However, at Time 3 three TAU participants agreed to questionnaire completion again, while only one participant in the other group did so. From Time 1 to Time 2, 75% of BTP+TAU participants were retained in the study. At the final follow up, all 12 of the participants who completed the intervention agreed to assessments visits. However, two participants requested to complete the questionnaires afterwards and their self-report data, with the exception of the EQ-5D-5L (49, 51), were subsequently lost in the post. Thus, full data from 62.5% of participants were available for analysis. In contrast, only 44% of TAU only participants remained engaged with the study at Time 2 but, with three participants returning to complete measures at Time 3, 61 % were retained in the end.

### Baby Triple P Engagement and Acceptability

Of the 16 participants allocated to receive BTP+TAU, 93% (*n* = 14) completed the four core sessions which were almost always delivered during their MBU admission. The first four sessions are essential, whilst the remaining four sessions are for parents to practice their skills and to problem solve; these final sessions are typically delivered *via* the telephone. The data revealed that 81% continued to engage with the intervention at Session 5 which then dropped to 75% for the remaining three sessions. Most participants would have been discharged from the MBU at this stage of the intervention. In total, 75% (*n* = 12) completed the intervention.

Ten of these 12 participants returned the *Client Satisfaction Questionnaire* (CSQ) (39) by post. Their overall mean score was 75.33, out of a total possible score of 91, which indicates high acceptability of the intervention (see Supplementary Table 3 for further details). Responses for individual domains indicate that participants particularly valued the quality of the programme. They were also satisfied with the overall delivery of the intervention and that they were particularly satisfied with the progress of their baby.

A more detailed analysis of the intervention's acceptability alongside the study procedures, based on interviews with study participants and MBU ward staff, will be reported elsewhere [see (34)].

### Serious Adverse Events and Contamination

No research related serious adverse events were recorded for any participants during the study period, suggesting that the parenting intervention was safe. All participants adhered to our requests of restricting any sharing of BTP learning and materials, such as the workbook, with their partners and/or family. No contamination issues were reported or observed.

### Secondary Outcome Measures

Of the participants who were retained in the study, completion of the assessment measures was very high for all five outcomes collected across the three assessments: MEQ (91%), DASS-21 (97%), PBQ (97%), BSI (94%) and EQ-5D-5L (100%). However, one participant in the BTP+TAU group completed the EQ-5D-5L only at the Time 2 assessment, but not the other self-report questionnaires. We did not identify any patterns in the missing data that indicated unacceptability of certain items or specific questionnaires. MBU staff completed observer-rated measures for 94% of participants at Time 1. However, their completion rate dropped to 20% at Time 2, which typically fell outside participants' average MBU admission of about 9 weeks, because MBU staff no longer had contact with study participants following discharge and could not report on their outcomes. Consequently, it was decided to not seek further information from staff at Time 3.

### Between-Group Effect Sizes

The mean total scores for the clinical outcome measures at baseline are presented in Table 4 and group differences following treatment and at follow-up are detailed in Table 5. Based on cut-off scores from the DASS (45), at baseline participants across both groups rated their symptoms of depression as severe, anxiety as extremely severe, and stress as moderate (see Table 4). Based on cut-off scores proposed by Brockington et al. (48), mean PBQ total scores as well as mean PBQ scores regarding impaired bonding indicate psychopathology in both groups. Although *t-*scores for BSI Global Severity Index suggest that this sample was below the cut-off score (i.e., *t-*score of 63) for identification of psychiatric disorders (61), participants' BSI scores appear to be higher than other clinical samples [e.g., (62)]. At baseline, MBU psychiatrists rated the participants as being moderately to severely ill [according to their CGI and BPRS assessments (54)]. Mothers allocated to the BTP+TAU group were also assessed as presenting with more risky behaviors related to baby care than the mothers from the TAU only group as assessed by Louis MACRO total scores and the Louis MACRO subscales scores regarding emotional care, parenting and mother's mental state (see Table 4).

**Table 4:** Self-reported and observer-related outcomes at baseline.

	**BTP +TAU (*n=* 16)**		**TAU only (*n =* 18)**		**Total (*n =* 34)**	
	***M***	***SD***	***M***	***SD***	***M***	***SD***
**Self-report measures**						
MEQ	52.44	10.58	52.78	10.81	52.62	10.54
DASS total	83.13	18.17	81.56	32.8	82.29	26.55
DASS depression	25.06	10.88	25	13.99	25.02	12.43
DASS anxiety	22.31	8.8	20.72	13.65	25.03	12.44
DASS stress	26.44	10.09	28.11	11.47	21.47	11.48
PBQ total	46.56	26.77	42	25.17	27.32	10.72
PBQ impaired bonding	18.81	12.85	19.22	12.3	19.03	12.37
PBQ rejection and pathological anger	13	8.07	11.56	8.07	12.24	7.98
PBQ infant focused anger	7.25	4.63	7.94	3.78	7.62	4.15
PBQ risk of abuse	2.19	3.97	3.28	3.98	1.5	3.51
BSI global Severity Index *t-*score (raw score)	58 (2.05)	6.66 (0.66)	60.12 (2.25)	10.61 (0.93)	59.09 (2.15)	8.85 (0.80)
BSI positive Symptom Total *t-*score (raw score)	59.38 (42.18)	7.92 (7.25)	60.88 (42.41)	11.22 (10.54)	60.15 (42.30)	9.64 (8.96)
BSI positive Symptom distress *t-*score (raw score)	55.81 (2.54)	8.06 (0.57)	56 (2.53)	10.69 (0.74)	55.91 (2.54)	9.36 (0.65)
BSI Somatisation	57.19 (1.62)	7.62 (0.75)	56.88 (1.69)	9.86 (1.09)	57.03 (1.68)	8.71 (0.92)
BSI obsessive-compulsive	59.5 (2.54)	7.45 (0.91)	60.47 (2.60)	9.42 (0.97)	60 (2.57)	8.4 (1.08)
BSI interpersonal sensitivity	58.5 (2.48)	9.97 (1.12)	60.41 (2.63)	9.93 (1.08)	59.48 (2.56)	9.84 (1.08)
BSI depression	57.38 (2.57)	9.11 (1.13)	56.12 (2.51)	10.43 (1.15)	56.73 (2.54)	9.68 (1.12)
BSI anxiety	56.19 (2.44)	5.38 (0.72)	55.71 (2.35)	8.6 (1.15)	55.94 (2.39)	7.11 (0.89)
BSI hostility	54.81 (1.45)	8.73 (1.06)	58.59 (1.85)	9.65 (1.22)	56.76 (1.66)	9.27 (1.15)
BSI phobic anxiety	59.56 (2.12)	6.83 (1.01)	62.35 (2.62)	8.54 (1.19)	61 (2.38)	7.77 (1.12)
BSI paranoid ideation	53.81 (1.58)	8.95 (1.04)	58.06 (2.04)	10.84 (1.31)	56 (1.82)	10.05 (1.19)
BSI psychoticism	55.5 (1.71)	6.35 (0.78)	57.94 (2.07)	11.92 (1.15)	56.76 (1.89)	9.56 (0.99)
**Staff-rated measures**						
Louis MACRO total	**16.97**	**1.32**	**18.08**	**1.42**	**17.6**	**1.47**
Louis MACRO safety	3.81	0.22	4.52	2.57	4.19	1.91
Louis MACRO physical care	3.75	0.22	3.84	0.29	3.8	0.26
Louis MACRO emotional care	**3.1**	**0.44**	**3.47**	**0.49**	**3.3**	**0.5**
Louis MACRO parenting	10.65	0.63	11.23	0.74	10.97	0.74
Louis MACRO infant characteristics	**3.41**	**0.4**	**3.59**	**0.52**	**3.51**	**0.47**
Louis MACRO mother's mental state	**3.11**	**0.41**	**3.35**	**0.65**	**3.24**	**0.56**
BPRS total	59.56	19.11	58.72	16.08	59.12	17.3
CGI severity	4.93	0.92	5	0.84	4.9	0.86

*17 participants were assessed for BSI scores in the TAU group; 15 participants were assessed for Louis MACRO scores in the BTP + TAU group. Values in bold indicate significant difference at p < 0.05*.

**Table 5:** Differences between groups after treatment.

**Post-intervention (10 weeks after baseline; Time 2)**	BTP + TAU (*N =* 11)*		**TAU only (*n =* 8)**		**Adjusted mean difference**	**SE**	**BCa 95% CI**		**Cohen's *d***
	***M***	***SD***	***M***	***SD***					
MEQ	64.45	7.92	60.63	13.60	4.27	4.04	−2.52	10.54	0.38
DASS total	51.45	29.73	74.25	29.69	−24.55	13.77	−52.02	5.96	−0.83
DASS depression^s^	14.55	12.36	22.25	12.85	−8.25	5.21	−17.38	1.04	−0.65
DASS anxiety^s^	15.27	10.13	16.88	11.23	−4.09	5.28	−13.75	8.73	−0.38
DASS stress^s^	19.82	10.45	29.38	9.40	−10.08	4.42	−18.16	−0.25	–1.02
PBQ total	20.27	23.44	29.00	27.21	−14.01	8.54	−32.20	4.39	−0.55
PBQ impaired bonding^s^	9.45	10.96	13.63	13.06	−5.30	3.67	−13.90	2.44	−0.44
PBQ rejection and pathological anger^s^	6.00	6.87	8.38	8.28	−3.53	2.16	−7.92	1.43	−0.46
PBQ infant focused anger^S^	3.91	3.51	5.25	4.06	−1.12	1.57	−4.48	2.36	−0.29
PBQ Incipient abuse^s^	0.91	3.02	1.50	3.51	0.71	0.62	−0.21	1.49	0.22
BSI global Severity Index (*t*-scores)	46.09	13.03	52.25	14.09	−7.32	4.48	−17.99	3.28	−0.73
BSI positive Symptom Total (*t*-scores)	47.55	13.70	53.88	14.82	−9.66	3.19	−16.71	−3.33	–0.92
BSI positive Symptom distress (t-scores)	42.73	10.21	50.75	12.65	−6.35	4.53	−14.30	2.65	−0.76
BSI somatisation^s^	48.18	9.83	49.50	11.67	−3.70	4.90	−12.02	4.66	−0.47
BSI obsessive-compulsive^s^	50.55	13.71	52.00	9.65	−3.23	5.61	−14.44	6.24	−0.36
BSI interpersonal^s^ sensitivity	49.55	10.43	57.75	9.93	−7.85	3.76	−14.90	−0.95	−1.04
BSI depression^s^	44.73	13.03	52.25	14.26	−7.98	4.66	−17.13	−0.04	−0.80
BSI anxiety^s^	43.91	11.11	49.25	11.35	−5.98	3.88	−13.59	2.33	−0.72
BSI hostility^s^	46.00	10.42	52.50	10.72	−6.79	3.78	−14.51	0.81	−0.87
BSI phobic anxiety^s^	50.91	11.71	56.50	14.02	−2.64	5.80	−11.45	4.57	−0.28
BSI paranoid ideation^s^	46.55	10.43	48.00	12.31	−4.48	3.32	−11.16	3.49	−0.54
BSI Psychoticism^s^	45.82	8.38	54.88	15.29	−9.87	2.87	−15.67	−4.11	−1.14
Clinical global impression	1.38	0.51	1.64	0.67	−0.23	0.25	−0.74	0.22	−0.48
**Six-month Follow-up (Time 3)**	**BTP** **+** **TAU (*****N** **=*** **10)**		**TAU only (*****n** **=*** **11)**		**Adjusted mean difference**	**SE**	**95% CI**		**Cohen's** ***d***
	***M***	***SD***	***M***	***SD***				
MEQ	64.90	11.32	61.80	9.77	2.38	0.18	0.36	0.98	0.22
DASS total	43.60	43.44	56.55	33.29	−18.09	16.28	−49.27	19.31	−0.47
DASS depression^s^	12.30	12.91	16.45	13.57	−5.91	5.99	−16.78	4.39	−0.45
DASS anxiety^s^	10.20	10.56	14.64	12.98	−7.48	5.65	−18.07	2.25	−0.63
DASS stress^s^	15.20	13.41	19.00	11.51	−5.89	5.31	−15.39	4.71	−0.47
PBQ total	20.50	17.89	34.36	32.32	−16.23	10.48	−35.31	4.19	−0.61
PBQ impaired bonding^s^	9.60	8.06	16.00	14.62	−5.67	4.24	−14.03	3.00	−0.47
PBQ rejection and pathological anger^s^	5.10	4.98	9.91	9.54	−5.30	2.78	−11.69	1.39	−0.69
PBQ infant focused anger^s^	4.80	4.52	5.64	4.63	−0.05	2.06	−4.58	5.16	−0.01
PBQ incipient abuse^s^	1.00	2.83	2.82	4.62	0.50	0.53	−0.47	1.79	0.13
BSI global Severity Index (t-scores)	47.30	16.83	49.82	12.95	−4.36	5.38	−14.25	7.65	−0.29
BSI positive Symptom Total (t-scores)	48.10	15.79	50.82	12.64	−3.29	4.39	−11.03	5.47	−0.23
BSI positive Symptom distress (t-scores)	48.10	19.30	48.36	12.69	−5.60	6.13	−17.74	9.34	−0.35
BSI somatisation^s^	49.60	12.02	51.27	12.02	−2.94	5.42	−12.77	7.26	−0.24
BSI obsessive-compulsive^s^	52.30	11.13	51.00	10.99	0.32	5.16	−8.54	8.54	0.03
BSI interpersonal sensitivity^s^	48.60	15.06	52.36	14.07	−2.99	5.37	−12.79	6.88	−0.21
BSI depression^s^	45.20	16.72	47.27	11.12	−4.06	5.06	−13.84	7.08	−0.29
BSI anxiety^s^	42.90	12.26	48.18	10.42	−8.40	4.08	−17.02	1.42	−0.74
BSI hostility^s^	47.90	15.18	50.64	10.41	−4.16	4.54	−12.49	4.48	−0.32
BSI phobic anxiety^s^	51.60	14.21	57.09	13.85	−3.48	5.88	−14.51	6.92	−0.25
BSI paranoid ideation^s^	47.30	14.39	47.82	12.59	−2.30	5.03	−12.80	11.07	−0.17
BSI psychoticism^s^	44.70	12.46	50.64	13.60	−5.64	4.82	−15.44	6.53	−0.43

*One participant at post-intervention assessment remained in the study but only completed the EQ-5D.

*^s^Indicates measure is a subscale. DASS stress difference at post-intervention significant at p = 0.04; BSI psychoticism difference significant at p = 0.02; BSI Positive symptom total significant at p = 0.01MEQ: Higher scores indicate higher maternal efficacy; DASS, PBQ AND BSI: lower scores indicate lower psychopathology; lower scores on the CGI indicate better mental health status*.

*10 participants from the TAU only group completed MEQ measure at six-month follow-up assessment*.

*Cohen's d: 0.2 = small effect, 0.5 = medium effect, 0.8 = large effect (63)*.

The adjusted mean differences between the groups indicate higher levels of improvement (i.e., higher scores in MEQ and lower scores on all other measures) for the intervention group than for the TAU only group at post-intervention (see Table 5). From baseline to post-intervention, large effect sizes (63) were observed for the DASS total scores as well as the DASS stress subscale scores. Improvements were also evident in terms of symptomatology: large effect sizes were noted for participant-completed BSI positive symptom Total and Distress scores, interpersonal sensitivity, depression, hostility and psychoticism. Although only evidenced by a medium effect size, improvements in mental state, as assessed by the psychiatry staff rated CGI, were also greater for the BTP+TAU group compared to the TAU only group. Improvements were also noted in mothers' perceptions of their parent-baby bond: medium effect sizes were noted for the overall PBQ total score as well as for the rejection and anger subscale both at post-intervention and six-month follow-up.

Although overall effect sizes seem to reduce from post-intervention to final follow-up, inspection of mean values for each group across assessment points indicate greater and sustained improvements in all available outcome measures for the BTP+TAU group compared to the TAU only group. However, the small sample size as well as the large range in confidence interval values could suggest imprecision of the effect of the differences between groups.

The potential benefits of the intervention were explored further: individual scores from self-reported measures were also assessed for clinical significance changes by calculating Jacobson and Truax's (64) reliable change index (RCI) from baseline to post-intervention and follow-up scores. We then summarized the number of participants with a reliable change indicating improvement (a score of > 1.96) in each treatment group. Results from this reliable change index analysis indicated that more participants in the BTP+TAU group showed clinically significant improvements from baseline to follow-up assessments in almost all of the self-reported measures compared to the TAU only participants (see Supplementary Table 4). The findings indicated that those in the intervention group improved more quickly from baseline to Time 2, especially in terms of self-reported symptoms of anxiety, depression and stress (DASS) and other symptoms (BSI), and these improvements were also evident from baseline to final assessment. Both groups gained in perceived maternal competence (MEQ) but only participants in the intervention group showed improvements in how they rated their overall bonding with their infants (e.g., 0% vs. 36% and 0% vs. 40% to Time 2 and Time 3, respectively).

### Economic Data

Utility values and QALYs derived from the EQ-5D-5L are summarized in Table 6. Over the whole follow-up period the intervention group had higher utility values on average than the TAU only group.

**Table 6:** Summary of EQ-5D data.

	**Mean (95% CI)**	
	**BTP+TAU (*n =* 16)**	**TAU** **(*n =* 18)**
Utility at Time 1	0.57 (0.48, 0.66) *n =* 16	0.55 (0.42, 0.68) *n =* 18
Utility at Time 2	0.70 (0.55, 0.85) *n =* 11	0.54 (0.26, 0.83) *n =* 8
Utility at Time 3	0.72 (0.55, 0.76) *n =* 10	0.63 (0.46, 0.79) *n =* 11
QALYs (baseline to week 26)	0.37 (0.31, 0.43) *n =* 8	0.27 (0.15, 0.40) *n =* 8
Net QALYs (95% CI)	0.10 (−0.02 to 0.22) *n =* 16	

The cost breakdown of delivering BTP in a MBU setting is summarized in Table 7. Training was delivered once in each Site over 3 days (assuming 7.5 h per day, this equates to 22.5 h). The total cost (including training) for Site 1 was estimated to be £443 per participant, based on an NHS Band 8a Clinical Psychologist delivering the training (£63/h, 64) and the 69 h delivering the intervention to 13 mothers. The total cost for Site 2 was estimated to be £822 per participant, based on an NHS Band 7 Occupational Therapist delivering the training (£53/h, 64) and the 24 h delivering the intervention to three mothers. The average cost across both sites was £514 per participant. Due to low recruitment at Site 2, the training was more expensive per participant who received the intervention at that MBU. Data regarding healthcare resource use, reported in Supplementary Table 5, suggest that there were differences between the groups, with greater resource use for the TAU only group for all services except for nurses. The healthcare resource use data were complete in almost all cases examined and the quality of the data were good.

**Table 7:** Summary of costs for training and intervention delivery.

	Cost of delivering intervention*	Cost of training**	**Total cost**	**Cost/** **participant**
Site 1 (*n =* 13)	£4,347	£1,418	£5,765	£ 443
Site 2 (*n =* 3)	£1,272	£1,193	£2,465	£ 822
Overall (*n =* 16)	£5,619	£2,611	£8,230	£ 514

*Total cost based on number of full hours of care provided (69 in Site 1, 24 in Site 2).

**Number of hours of training (3 days at 7.5 per day = 22.5 h) x unit cost of trainer and trainee's time.

The details of the index MBU admission were recorded for 22 out of 34 participants. The mean length of stay was 62 days (95% CI 39 to 85; *n* = 10) in the TAU group and 64 days (95% CI 47 to 82; *n* = 12) in the BTP+TAU group. The unit cost per MBU bed per night was £729 (65). The mean cost of the index MBU admission was £46,778 (95% CI 34,084 to 59,471) in the intervention group and £45,417 (95% CI 28,731 to 62,103) in the control group. Two participants were re-admitted to an MBU following index admission (both from the TAU group), totalling an additional 37 days of inpatient care in an MBU (costing £26,973); this may be a key driver of cost.

## Discussion

### Feasibility, Acceptability and Satisfaction Indicators

This is the first feasibility trial to evaluate the feasibility and acceptability of a structured parenting intervention like Baby Triple P in mothers with severe mental health difficulties. The findings indicate that mothers who were MBU inpatients found the intervention and its delivery in this setting acceptable. In addition, the individually randomized trial design, including the randomization procedure, was found to be feasible to be scaled up to a fully powered RCT to evaluate the effectiveness and cost-effectiveness of BTP in this setting. However, recruitment was lower than anticipated due to lower MBU admissions so the optimal approach to recruitment may need to be revisited.

In terms of outcome measure suitability, the high rates of participants answering all items as well as the low number of items being omitted suggest that outcome measures were acceptable and user-friendly. Furthermore, no specific patterns of items being left out were identified which indicates that the questions raised in the measures were acceptable for this population. Overall, the excellent rate of data completion (of >95% across all three assessment points) could be an indicator of our patient-reported outcomes (i.e., self-reported measures) being suitable for gauging differences in a future trial. Completion of observer-rated measures by MBU staff was also excellent during participants' admission for the first assessment time point (94%). However, at subsequent assessment times MBU staff could not complete those measures consistently because participants had been discharged. In a future RCT, the possibility of including observer-rated measures that can be administered by research staff should be considered alongside a stronger request for all self-reported measures to be completed during the follow-up appointments, even if online. Online or remote data collection has increased in use over the last 2 years to minimize infection risks; hence, tried and tested methods could be used in a future trial.

Although 100% of women admitted during the recruitment window were screened for study eligibility, the available pool to recruit from was lower than expected, potentially reflecting the early stages of the transformation of the perinatal mental health service provision in the UK (66). Furthermore, there was a difference in the recruitment rate between the two sites: the site with lower recruitment admitted more women with complex problems during the recruitment window and so they could not be included in the study. In addition, an increase in complex problems often meant longer admissions and a slower turnover of potential participants being admitted to that MBU. However, the diagnoses reported for this total sample are similar to those reported in previous MBU surveys in the UK (37, 67–69). Finally, although our inclusion criteria were relatively broad, women had to continue to reside on the ward for at least another week after expressing interest in the study to be eligible. Thus, only about 41% of all women admitted were eligible for study inclusion. Recruitment from more MBUs and possibly the inclusion of participants from recently developed perinatal CMHTs (66) would ensure better recruitment opportunities in future trials, potentially an even more diverse ethnic representation. The inclusion of participants who are not proficient in English should also be considered in any future study to test interventions for diverse groups of parents with mental health problems. In addition, the involvement of much younger mothers (≥16 years) could also be considered. As seen in this study, three mothers were eligible for study inclusion, but their relatively quick improvements meant they were discharged from the MBU much faster than originally anticipated by staff. If a future trial recruited from inpatient and outpatient perinatal mental health services, the timeframe of service use could be revised substantially, given the expectation that women discharged from MBUs are seen within relevant local perinatal mental health services.

Our intervention retention rate of 93% after the four core sessions of BTP was excellent and higher for our sample of mothers with severe mental health problems compared to a sample of mothers with postnatal depression [intervention retention rate of 85%; (30)] or a sample of non-psychiatric, healthy parents of premature babies [80%; (29)]. The completion of those crucial core Triple P sessions could be seen to constitute programme completion, because the remaining four sessions are designed for parents to practice their learnt parenting skills. Our retention rate of 75% at the end of the intervention was also higher compared to parents of premature babies [66%; (29)] and first-time, healthy mothers [40%; (28)], but not as promising as that of 85% for mothers with postnatal depression (30). Overall, our intervention attrition rate of 13% compares positively with attrition rates from Triple P interventions for parents of older children (>2 years) as attrition rates across several studies reached 19.5% [see meta-analysis by Nowak and Heinrichs (70)].

### Clinical Outcome Indicators

Although this study was not powered to detect statistically significant differences between the treatment groups, we identified signals of effectiveness. At Time 2 following the intervention, greater improvements were noted for the BTP+TAU group than the TAU group on all of the measures. The biggest differences (i.e., large effect sizes) were for participant-reported depression, anxiety and stress (i.e., DASS-21 scores) and for positive symptom total scores on the BSI which measures psychiatric symptom presence and severity. At Time 3 the between group differences had attenuated for almost all measures except the PBQ which evaluates the mother-baby bond and relationship. Considering that there is evidence to suggest that early remission of postnatal psychiatric difficulties can mitigate the effects of maternal SMI on child development (71), the results from the present study could be worth exploring in a full RCT. These findings may also suggest that the expected mechanism of action of this parenting intervention in relation to strengthening the mother-infant relationship may be more evident over a longer period. Accurately capturing the changes in the parent-infant relationship across a longer time period, potentially without relying solely on self-report measures [e.g., (72)], would be crucial for any future trial.

### Economic Outcome Indicators

The quality and completeness of data from the resource use and EQ-5D-5L questionnaires were good. There was a net QALY gain in the BTP+TAU group compared with the TAU only group, which may signal additional health benefits associated with BTP. This difference was not statistically significant, but the sample size was small and the study was not powered to detect differences in QALYs. The cost to deliver BTP was modest; however, it may be possible to explore whether BTP could be delivered over fewer sessions or by lower salaried professionals without reducing the fidelity of the intervention. The resource use questionnaire is well designed for data collection when administered in an interview format by field researchers as done here. It may be necessary to modify the design if a different mode of completion was used in a full RCT. A key driver of healthcare costs among the study participants was MBU readmission. Two participants from the TAU only group were readmitted to their MBUs which was associated with a cost of £26,973. None of the participants randomized to BTP+TAU was readmitted. If the addition of this parenting intervention to usual care was associated with a lower likelihood of readmission to a MBU this would be an important outcome. However, given the small number of participants included in this analysis, it is possible that this was observed by chance.

### Challenges of Undertaking This Study in a MBU Setting and Limitations

Although acute psychiatric wards have been found to be complex and challenging environments for patients as well as staff, staff supported the consent, recruitment and other study procedures at both MBUs. The longer admissions for the women recruited to this study may indicate more complex mental health presentations, but it is important to note that those women allocated to receive the intervention engaged extremely well, especially during their inpatient admission. However, other challenges should be acknowledged. Two MBUs, comparable in terms of size and other relevant factors, were used to enhance the generalisability of the findings, but only one site almost met the anticipated recruitment rate whilst the other one did not. During this study admissions to the lower recruitment site varied and that MBU was not always operating at full capacity. Over the last few years MBUs in the UK have been part of a changing service landscape with the development of perinatal mental health services including community service provision. It is possible that those developments impacted one MBU more than the other.

The collection of staff-rated measures proved challenging after participants were discharged from the MBU and several measures could not be included. Some of these measures were chosen because they were routinely collected outcomes at both MBUs during the study planning phase (e.g., HONOS and LouisMACRO). For any future trial, observer-rated measures should be chosen carefully so that research staff could complete these. The transformation of perinatal mental health services in the UK may ensure that women will be referred to perinatal CMHTs following MBU-discharge which means that routine outcome measures used within these services could be considered and the woman's care coordinator could be asked to assist with the completion of measures. It would also be sensible to extend the trial to include such services in order to omit the exclusion criterion of ward presence for a period of seven days following expressing an interest in the study; we had to exclude three participants for this reason. Furthermore, due to its established training structure, the intervention can easily be offered by various members of an inpatient or outpatient perinatal mental health team, including nursery nurses. BTP was developed for the infant's parents so delivery does not have be restricted to the mother but can successfully be offered to fathers as well as other significant others in the case of single parents. As many countries may have different criteria for the admission of a mother to a psychiatric unit, sometimes without the ability for a joint mother and baby admission, it would be useful to explore the benefits of this type of parenting interventions in other countries as an adjust to the mother's usual mental health care. Finally, the inclusion of a video-based assessment of the parent-infant interaction and relationship should be considered to assess potential attachment behaviors shown by the infant and to have a more objective measure of the parent-child interaction, rated by trained and accredited coders. The intervention clearly supported mothers bonding with their infants and this aspect warrants further investigation, especially in relation to the infant's psychological development and mental wellbeing.

When interpreting the current findings, some limitations need to be considered. The study sample size was relatively small and admission rates to both MBUs were lower than anticipated. Uptake and engagement rates of mothers need to be explored further in an outpatient mental health setting. Whilst no contamination issues were reported by participants or observed by MBU staff, this possibility remains. However, as the prevalence of maternal mental illness is reportedly rising (73), there is a need to explore the benefits of a range of interventions that can improve the mental health of mothers as well as their bonding with their infants.

### Conclusions

This preliminary study indicates that a parenting intervention like Baby Triple P can be delivered and implemented successfully in acute psychiatric inpatient settings like a Mother and Baby Unit to service users who appeared to benefit from what the intervention has to offer to them and their infants. We identified that this intervention and our study procedures could be delivered safely. There was good retention in both treatment groups and an exceptionally high level of completion of self-report outcome measures. Some modifications may need to be made to the collection of observer-rated outcomes but overall the findings suggest that the study procedures are feasible for a future large-scale trial of a structured parenting intervention that would complement other therapeutic approaches perfectly and could easily be implemented into existing perinatal mental health services.

## Data Availability Statement

The raw data can be made available by the authors on request.

## Ethics Statement

This trial was supported by the NIHR Research for Patient Benefit Programme (NIHR RfPB, grant number PB-PG-1014-3505) and sponsored by the University of Manchester. Study approvals were granted by the NHS National Research Ethics Service (NRES) *via* the Northwest–Greater Manchester South Research Ethics Committee (REC) (16/NW/0510), the Health Research Authority (HRA) (IRAS project number 188486, protocol number 16233) and the Research and Innovation departments of both NHS trusts overseeing the two participating MBUs (e.g., Greater Manchester Mental Health NHS Foundation Trust and Birmingham and Solihull Mental Health NHS Foundation Trust). The patients/participants provided their written informed consent to participate in this study.

## Author Contributions

AW conceived of the idea and developed it into a proposal with the help of RC, RE, PB, EC, and KA. RE led on the statistical analysis and EC on the health economic evaluation. KC and PD supported trial coordination, while HR and PG acted as research assistants, involved in data collection or data analysis. All authors contributed to the development of this manuscript and approved its publication.

## Funding

This feasibility trial represents independent research, which was funded by the National Institute for Health Research (NIHR) under its Research for Patient Benefit (RfPB) Programme (PB-PG-1014-35059). The work was also supported by the Greater Manchester Mental Health NHS Foundation Trust (GMMH) and the Birmingham and Solihull Mental Health NHS Foundation Trust (BSMH). The Open Access publication was made possible by the support of the University of Manchester. RE is supported by the NIHR Biomedical Research Centre at South London and Maudsley NHS Foundation Trust and King's College London and an NIHR Research Professorship (NIHR300051).

## Author Disclaimer

The views expressed are those of the authors and not necessarily those of the NHS, the NIHR or the Department of Health.

## Conflict of Interest

The authors declare that the research was conducted in the absence of any commercial or financial relationships that could be construed as a potential conflict of interest.

## Publisher's Note

All claims expressed in this article are solely those of the authors and do not necessarily represent those of their affiliated organizations, or those of the publisher, the editors and the reviewers. Any product that may be evaluated in this article, or claim that may be made by its manufacturer, is not guaranteed or endorsed by the publisher.
