# Supplementary material for: A Feasibility Randomized Controlled Trial of a Parenting Intervention Offered to Women With Severe Mental Health Problems and Delivered in a Mother and Baby Unit Setting: The IMAgINE Study Outcomes
Source: Front Psychiatry. 2022 May 16;13:815018. doi: 10.3389/fpsyt.2022.815018 (PMC9149174; doi:10.3389/fpsyt.2022.815018)
Supplement: Supplementary file 1 [file Table_1.DOCX]

| **Supplementary Table 1: Diagnosis by consultant psychiatrist on admission** | | | |
| --- | --- | --- | --- |
|  | **BTP+TAU (n=16)** | **TAU Only (n=18)** | **Total (n=34)** |
| **Postpartum psychosis** | 6% (n= 1) | 16% (n= 3) | 12% (n= 4) |
| **Bipolar disorder** | 19% (n= 3) | 11% (n= 2) | 15% (n= 5) |
| **Depression with psychotic features** | 6% (n= 1) | 16% (n= 3) | 12% (n= 4) |
| **Depression** | 31% (n= 5) | 11% (n= 2) | 21% (n= 7) |
| **Anxiety (including GAD and PTSD)** | 12% (n=2) | 0% (n=0) | 6% (n=2) |
| **Anxiety (including GAD, PTSD and OCD) and affective disorders** | 12% (n=2) | 16% (n=4) | 21% (n=6) |
| **Personality disorder and affective disorders** | 6% (n= 1) | 16% (n= 4) | 15% (n= 5) |
| **Schizophrenia** | 6% (n= 1) | 0% (n= 0) | 2% (n= 1) |
| Note: percentages calculated based on group sample and total sample | | | |

**Supplementary Table 2: Changes made to the initial protocol**

| **Staff interviews - inclusion criteria** | Semi-structured interviews were conducted with MBUs wards to explore facilitators and barriers to implement study in ward settings eligibility criteria excluded junior doctors. However, these criteria were modified since junior doctors were involved in many aspects of conducting the study. |
| --- | --- |
| **Recruitment extension** | Recruitment was extended from 15 to 18 months because there were delays in the recruitment start date at Site 2 and admission rates were lower than anticipated before the start of the study. |
| **Target number of eligible participants** | Initially, we planned to recruit 60 participants. However, this target had to be revised as admission rates were lower than anticipated. We adjusted the target number to 38 participants based on information on admission rates at the start of the study. |
| **Administration of outcome measures** | HoNOS was completed at time 1 during the preparation phase of the study, which led us to include it as an outcome measure. However, when the study commenced, Site 1 stopped administering HoNOS as an outcome measure routine, therefore, it was not possible to include this measure in the assessment. |
| **Intention to treat approach** | It was initially anticipated that an intention to treat approach was going to be used to handle missing values. As the amount of available data was low, analyses were conducted using all available values rather than performing multiple imputation. |
| **Exclusion of NHS Trust as a covariate in quantitative analysis** | We initially planned to conduct the analysis of the efficacy outcomes including NHS as a covariate along with Group and Baseline measures. However, given the low number of participants recruited at Site 2, we decided it was not appropriate to include NHS Trust in the analysis. |
| **Analysis of observer-rated measures** | Most of the participants were discharged from the MBUs before post-intervention assessment, which prevented the staff from administering these measures as it was initially planned in the protocol. For this reason, linear regression analyses were not conducted for observer-rated outcomes. |

| **Supplementary Table 3: Client satisfaction ratings (7-point scale) for women allocated to Baby Triple P** | | |
| --- | --- | --- |
| **Item** | **Client satisfaction questionnaire**  **(*n* = 10)** | |
|  | ***M*** | ***SD*** |
| Quality of service | 6.11 | 0.79 |
| Baby Triple P provided the help sought | 5.78 | 0.88 |
| Baby Triple P met child’s needs | 5.67 | 1.43 |
| Baby Triple P met parental needs | 5.67 | 0.84 |
| Satisfied with amount of help | 6.11 | 0.79 |
| Programme has helped with child behaviour | 5.89 | 1.14 |
| Programme has helped deal with family problems | 5.78 | 0.99 |
| Programme has helped with partner relationship | 4.78 | 1.48 |
| Overall satisfaction with programme | 6.22 | 0.82 |
| Would do Triple P again if needed | 5.67 | 0.97 |
| Programme skills can be applied to other family members | 5.89 | 0.94 |
| Child’s development at this point | 5.44 | 0.85 |
| Satisfaction with child progress | 6.33 | 0.97 |
| Total score (possible total score of 91) | 75.33 | 9.39 |

| **Supplementary Table 4: Overview of clinically significant changes** | | | | | | | | |
| --- | --- | --- | --- | --- | --- | --- | --- | --- |
|  | **Baseline to post-intervention** | | | | **Baseline to 6-month follow-up** | | | |
|  | **BTP+TAU (n=11)** | | **TAU**  **(n=8)** | | **BTP+TAU (n=10)** | | **TAU**  **(n=11)** | |
|  | **n** | **%** | **n** | **%** | **n** | **%** | **n** | **%** |
| **MEQ** | 8 | 73% | 5 | 63% | 7 | 70% | 9 | 82% |
| **DASS Total** | 8 | 73% | 2 | 25% | 7 | 70% | 6 | 55% |
| **DASS Depression** | 7 | 64% | 2 | 25% | 7 | 70% | 5 | 45% |
| **DASS Anxiety** | 7 | 64% | 3 | 38% | 7 | 70% | 5 | 45% |
| **DASS Stress** | 9 | 82% | 2 | 25% | 7 | 70% | 5 | 45% |
| **PBQ Total** | 4 | 36% | 0 | 0% | 4 | 40% | 0 | 0% |
| **PBQ Bonding** | 3 | 27% | 0 | 0% | 3 | 30% | 0 | 0% |
| **PBQ Rejection** | 3 | 27% | 0 | 0% | 3 | 30% | 1 | 9% |
| **PBQ Anger** | 2 | 18% | 1 | 13% | 2 | 20% | 2 | 18% |
| **PBQ Abuse** | 1 | 9% | 3 | 38% | 2 | 20% | 3 | 27% |
| **BSI Global severity index** | 7 | 64% | 1 | 13% | 7 | 70% | 4 | 36% |
| **BSI Positive symptom total** | 6 | 55% | 0 | 0% | 0 | 0% | 0 | 0% |
| **BSI Positive symptom distress** | 6 | 55% | 1 | 13% | 5 | 50% | 3 | 27% |
| **BSI Somatisation** | 6 | 55% | 0 | 0% | 4 | 40% | 3 | 27% |
| **BSI Obsessive-compulsive** | 5 | 45% | 2 | 25% | 6 | 60% | 4 | 36% |
| **BSI Interpersonal sensitivity** | 3 | 27% | 0 | 0% | 6 | 60% | 2 | 18% |
| **BSI Depression** | 5 | 45% | 2 | 25% | 5 | 50% | 4 | 36% |
| **BSI Anxiety** | 3 | 27% | 1 | 13% | 4 | 40% | 2 | 18% |
| **BSI Hostility** | 3 | 27% | 1 | 13% | 5 | 50% | 1 | 9% |
| **BSI Phobic anxiety** | 4 | 36% | 2 | 25% | 4 | 40% | 2 | 18% |
| **BSI Paranoid ideation** | 3 | 27% | 0 | 0% | 3 | 30% | 0 | 0% |
| **BSI Psychoticism** | 2 | 18% | 0 | 0% | 3 | 30% | 1 | 9% |

**Supplementary Table 5. Summary of utility by treatment group**

|  | **Mean (95% CI)** | |
| --- | --- | --- |
|  | **BTP+TAU intervention**  **(n=16)** | **TAU**  **(n=18)** |
| Utility at time 1 | 0.57 (0.48, 0.66)  n=16 | 0.55 (0.42, 0.68)  n=18 |
| Utility at time 2 | 0.70 (0.55, 0.85)  n=11 | 0.54 (0.26, 0.83)  n=8 |
| Utility at time 3 | 0.72 (0.55, 0.76)  n=10 | 0.63 (0.46, 0.79)  n=11 |
| QALYs (baseline to week 26) | 0.37 (0.31, 0.43)  n=8 | 0.27 (0.15, 0.40)  n=8 |
| Net QALYs (95% CI) | 0.10 (-0.02 to 0.22)  n=16 | |

|  | **Unit cost (per hour)** | **Cost of delivering intervention*** | **Cost of training**** | **Cost/participant** |
| --- | --- | --- | --- | --- |
| **Site 1 (n=13)** | £63 | £4,347 | £1,418 | £ 443 |
| **Site 2 (n=3)** | £53 | £1,272 | £1,193 | £ 822 |
|  | Total delivery cost | £5,619 | £2,611 |  |
|  | Total cost** | | £8,230 |  |
|  | Cost/participant*** | | £ 514 |  |

*total cost based on number of full hours of care provided (69 in Site 1, 24 in Site 2)

**training cost = number of hours of training (3 days at 7.5 per day = 22.5hrs) x unit cost of trainee's time

***total overall cost divided by total number of participant (n=16)
